# Researching the acceptability of using Skype to provide Speech and Language Therapy

**Published:** 2012-06-15

**Authors:** Rebecca Alison Matthews, Bencie Woll, Mike Clarke

**Affiliations:** Speech Sort Ltd., Independent Speech and Language Therapy, UK; University College London, UK; University College London, UK

**Keywords:** tele-technology, Skype, speech and language therapy, tele-therapy

## Abstract

In the current economic climate, whilst the demand for health services, including Speech and Language Therapy (SLT) continues to rise, there is pressure to reduce health service budgets, Tele-technology—the use of tele-communication technology to link patient and clinician remotely—could potentially provide a solution to meeting the demand for SLT with reduced resources. However, only a few SLT services in the United Kingdom (UK) have reported on using tele-technology to provide their service (Howell, Tripoliti and Pring, 2009; Styles, 2008; McCullough, 2001; Katsavarus, 2001).

In 2002 the American Speech and Hearing Association (ASHA) surveyed its members on their experience and views of using tele-technology and specifically video-conferencing to provide an SLT service. The analysis of the responses identified five areas of concern—lack of professional guidelines, limited evidence of clinical efficacy, disruption and problems managing the technology, change in the interaction and loss of rapport as well as anticipated, additional costs to provide the service.

The study reported here set up an SLT service using the desktop videoconferencing system, Skype, in an independent SLT practice based in the UK. Data were collected to evaluate the acceptability of the clinical sessions, the technology, the quality of interaction and costs of an SLT service using Skype. Eleven participants aged between 7 and 14 years with varying therapy needs took part. Each received a mix of face-to-face (F2F) and Skype SLT over the ten session trial period. Data were collected for every session using a report card; adults supporting the children were asked for their views using a questionnaire at the beginning and end of the trial; the child participants were interviewed after the trial period was over; one F2F and one Skype session was video recorded for each participant; work activity was recorded along with identifiable costs of F2F and Skype SLT sessions.

A total of 110 session report cards, 24 questionnaires, 10 interviews and 16 hours of video recording were analysed. The responses from interviews and questionnaires were compared with the discourse analysis of the session video recordings and session report cards. A greater percentage of goals were completed in the Skype sessions. Whilst there were breaks in internet connection, this did not reduce the number of goals achieved. The ratio of turn moves and utterance functions were the same for therapist and child in both session formats; the only difference identified was the increased use of requests for objects/action in the F2F session. The discourse analysis supported the participant observations that the children worked harder and focused better in the Skype sessions. The presence of clarifications and the reduced numbers of interruptions were in line with other research observations that the speakers were able to judge the mood of the conversation partner in remote interaction. Costs of Skype SLT were lower than equivalent F2F sessions. The combination of positive findings suggests that Skype SLT sessions are potentially acceptable and are likely to have a useful role to play in service provision.

